# Protective Effects of Sodium-Glucose Transporter 2 Inhibitors on Atrial Fibrillation and Atrial Flutter: A Systematic Review and Meta- Analysis of Randomized Placebo-Controlled Trials

**DOI:** 10.3389/fendo.2021.619586

**Published:** 2021-03-19

**Authors:** Daobo Li, Yingying Liu, Tesfaldet Habtemariam Hidru, Xiaolei Yang, Yunsong Wang, Cheng Chen, Ka Hou Christien Li, Yuqi Tang, Yushan Wei, Gary Tse, Yunlong Xia

**Affiliations:** ^1^ Department of Cardiology, First Affiliated Hospital of Dalian Medical University, Dalian, China; ^2^ Faculty of Medicine, Newcastle University, Newcastle, United Kingdom

**Keywords:** sodium-glucose transporter 2 inhibitors, dapagliflozin, atrial fibrillation, atrial flutter, prevention

## Abstract

**Background:**

Hyperglycemia is associated with an increased risk of developing atrial fibrillation (AF) and atrial flutter (AFL). Sodium-glucose transporter 2 inhibitors (SGLT2i) have been reported to prevent AF/AFL in some studies, but not others. Therefore, a meta-analysis was performed to investigate whether SGLT2i use is associated with lower risks of AF/AFL.

**Methods:**

PubMed, Scopus, Web of Science, Cochrane library databases were searched for randomized placebo-controlled trials comparing SGLT2i and placebo.

**Results:**

A total of 33 trials involving 66,685 patients were included. The serious adverse events (SAEs) of AF/AFL occurrence were significantly lower in the SGLT2i group than the placebo group (0.96% vs. 1.19%; RR 0.83; 95% CI 0.71–0.96; P = 0.01; I^2^ 25.5%). Similarly, the SAEs of AF occurrence was significantly lower in the SGLT2i group (0.82% vs. 1.06%; RR 0.81; 95% CI 0.69–0.95; P = 0.01; I^2^ 10.2%). The subgroup analysis showed that the reduction in AF/AFL was significant only for dapagliflozin (1.02% vs. 1.49%; RR 0.73; 95% CI 0.59–0.89; P = 0.002; I2 0%), but not for canagliflozin (1.00% vs 1.08%; RR 0.83; 95% CI 0.62–1.12; P = 0.23; I^2^ 0%), empagliflozin (0.88% vs 0.70%; RR 1.20; 95% CI 0.76–1.90; P = 0.43; I^2^ 0%), ertugliflozin (1.01% vs 0.96%; RR 1.08; 95% CI 0.66–1.75; P = 0.76; I^2^ 0%), and sotagliflozin (0.16% vs 0.10%; RR 1.09; 95% CI 0.13–8.86; P = 0.93; I^2^ 0%).

**Conclusions:**

SGLT2i use is associated with a 19.33% lower SAEs of AF/AFL compared with the placebo. Dapagliflozin users had the lowest SAEs of AF/AFL incidence. Further studies are needed to determine whether canagliflozin, empagliflozin, ertugliflozin, and sotagliflozin similarly exert protective effects against AF/AFL development.

## Introduction

Patients with hyperglycemia such as type 2 diabetes mellitus (T2DM) are at increased risks of developing arrhythmias such as atrial fibrillation (AF) and atrial flutter (AFL) (1–3). Hyperglycemia and fluctuations in blood glucose levels can contribute to cardiac electrophysiological and structural remodeling, particularly in the atria (4, 5). Cardiovascular comorbidities such as heart failure (HF) also play a significant role in increasing AF/AFL incidence (6, 7). Even with optimal medical treatment, patients with T2DM may nevertheless go on to develop AF/AFL (8). Given that AF/AFL is associated with adverse outcomes such as HF and stroke (9), there is a need to identify treatment options that can prevent their development.

The underlying pathophysiology linking T2DM to AF predominantly favors the theory involving the generation of reactive oxygen species (ROS) secondary to hyperglycemia (10), which can lead to atrial cardiomyopathic changes (11, 12). While many interventions ranging from weight loss, angiotensin-converting enzyme inhibitors (ACEIs)/angiotensin receptor blockers (ARBs) to catheter ablation are used to prevent or treat AF, the diabetic medications can also protect against AF development (9, 13).

The sodium-glucose transporter inhibitor (SGLT2i) is a new class of anti-diabetic agents and works by inhibiting the reabsorption of sodium and glucose by the kidneys (14). Their use has been associated with a lower incidence of adverse events including all-cause mortality, cardiovascular mortality, HF, and AF (15–18). In clinical practice, SGLT2i is currently recommended for T2DM as a second- or third-line agent following inadequate glycemic control using metformin and/or sulphonylureas (19–21).

Animal studies have demonstrated that SGLT2i could reduce the oxidative stress in cardiomyocytes, which in turn reverses myocardial structural/electronic remodeling (22, 23). The post-hoc analysis of the DECLARE-TIMI 58 trial confirmed that dapagliflozin has a lower incidence of AF over placebo, indicated the potential benefit of SGLT2i in preventing AF/AFL (24), as confirmed by subsequent meta-analyses (25, 26). Recent studies have reported beneficial effects of SGLT2i in preventing atrial remodeling even in non-diabetic conditions. Therefore, we conducted this systematic review and meta-analysis of placebo-controlled trials to investigate the clinical effectiveness of SGLT2i in AF/AFL prevention among patients with or without T2DM.

## Methods

### Search Strategy and Data Sources

An electronic search of PubMed, Scopus, Web of Science and Cochrane library databases was conducted until 3rd December, 2020 using searching terms and related items including keywords “sodium-glucose transporter 2 inhibitors,” “sodium-glucose cotransporter 2 inhibitors,” “SGLT2i,” “dapagliflozin,” “BMS 512148,” “empagliflozin,” “BI 10773,” “canagliflozin,” “JNJ 28431754,” “tofogliflozin,” “CSG452,” “luseogliflozin,” “TS071,” “ipragliflozin,” “ASP1941,” “sotagliflozin,” “LX4211,” “ertugliflozin,” and “PF04971729.” The search algorithm is shown in **Table S1** in the **Supplementary Appendix**.

### Inclusion and Exclusion Criteria

The inclusion criteria were: (1) randomized placebo-controlled trials registered in ClinicalTrials.gov comparing SGLT2i with matching placebo including recorded AF/AFL outcomes; and (2) involving adult patients (>18 years of age) and iii) published in English language. The exclusion criteria were: (1) non-randomized placebo-controlled trials; (2) lack of information on the occurrences of AF/AFL; and (3) animal studies. This meta-analysis was performed under the recommendation of the preferred reporting items for systemic review and meta-analyses (PRISMA) guidelines, and the retrieved data were reviewed and approved by the principal investigator.

### Study Selection and Outcome Identification

All the studies were independently identified, reviewed, and screened by two authors (YL and YWa) based on their titles and abstracts to identify eligible studies. The authors performed a full-text review of the selected articles, and data were summarized in a prespecified spreadsheet in Microsoft Excel. All potentially relevant reports were retrieved as complete manuscripts and assessed for compliance with the inclusion criteria. Decisions of inclusion and exclusion were resolved by consensus between the reviewers. Agreement between reviewers for study selection was examined using the Kappa statistic. A third reviewer (DL) addressed disagreements concerning study inclusion. Citations matching inclusion criteria were included in the final analysis.

### Data Extraction and Quality Assessment

The characteristics of the studies (first author, year of publication, study design, and inclusion criteria) were extracted into an Excel file after identifying all relevant articles. Demographic and baseline patient characteristics were collected from all included trials. The Excel file contained the total number of participants in each trial, the number of participants who were in SGLT2i during the period of the trial, and the corresponding total number of AF/AFL occurrences.

AF/AFL outcomes were extracted from the eligible studies. We further retrieved relevant clinical data through clinicaltrials.gov, www.who.int/ictrp, or by browsing supplementary materials. The individual study outcomes were reported according to whether SGLT2i lowers serious adverse events (SAEs), particularly the total number of AF/AFL events during follow-up. The risk of bias of included trials was assessed through the Cochrane Collaboration’s tool for assessing the risk of bias by two reviewers (YL and YWa) independently. Each domain was assigned low, unclear, or high risk of bias. Since the data used in the meta-analysis derive from previously published studies, the approval of the Institutional Review Board was not necessary, and the analytical methods will not be made available to other researchers for the reproduction of the findings or replication of the procedure.

### Outcomes

The outcomes of our meta-analysis were SAEs of AF or AFL incidence for SGLT2i as a treatment group versus matching placebo. AF and AFL were defined as reported SAEs among included trials. The source of data on SAE were their Supplementary Materials of the publications for three trials (NCT01730534 [DECLARE-TIMI58 trial], NCT03036150 [DAPA-CKD trial], and NCT01986881). For the remaining 30, we obtained the information from clinicaltrials.gov.

### Statistical Analysis

Data were summarized using descriptive statistics including proportions for categorical variables. The overall risk ratio (RR) of efficacy outcomes was estimated using a Mantel-Haenszel random-effects model. The pooled SAEs of AF/AFL incidence events for the SGLT2i group and placebo group were also computed. The I^2^ statistics were assessed to quantify the heterogeneity in RR across the studies. I^2^ statistics <25%, 25–75%, and >75% were used to represent low, moderate, and a high degree of heterogeneity, respectively, at Cochrane *P*-*value ≤*0.05. Funnel plots were used to assess publication bias. All statistical analyses were conducted with the Review Manager (RevMan, version 5.3, The Cochrane Collaboration, Copenhagen, Denmark).

## Results

### Literature Search Results

**Figure 1:**
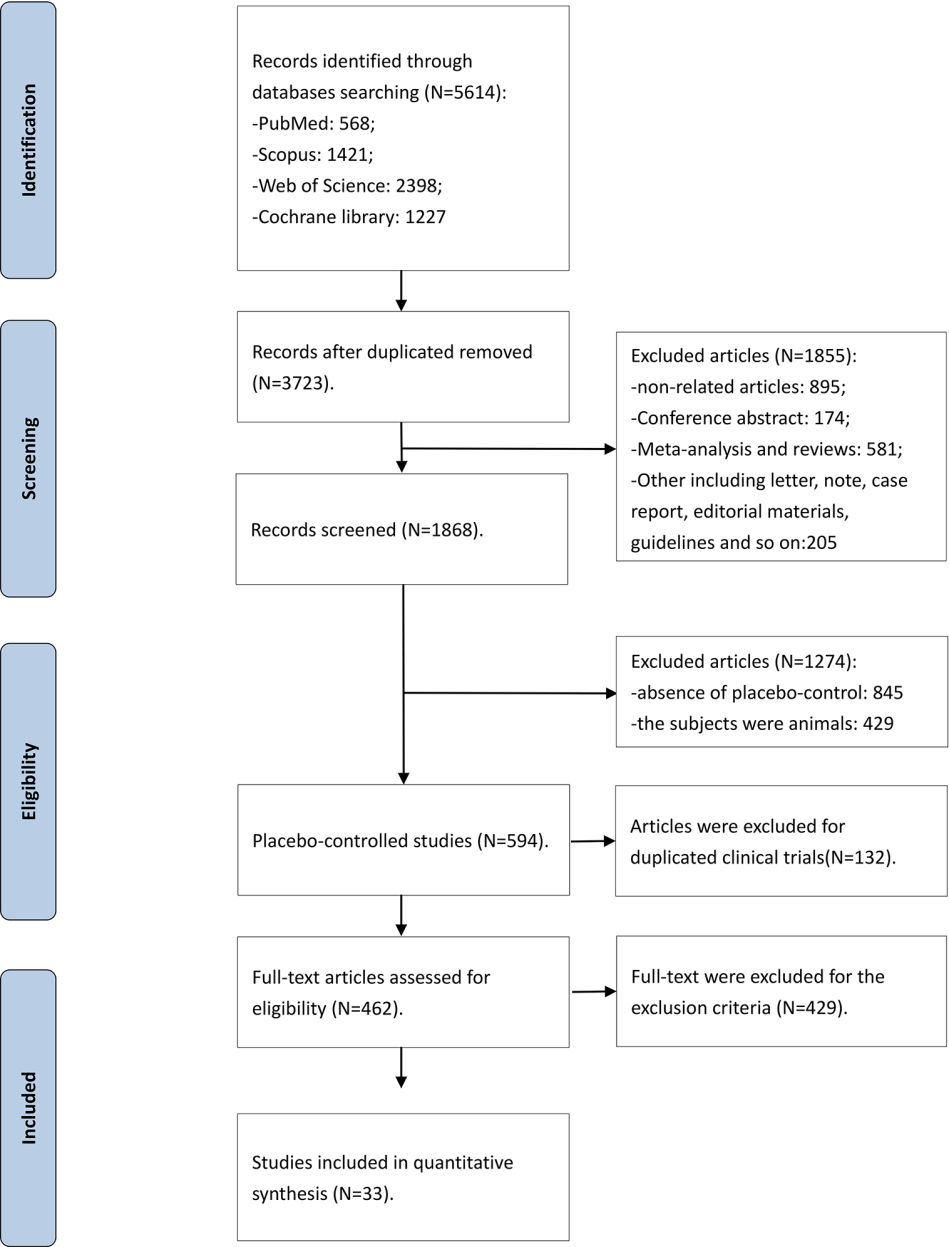
The flow diagram.

The flow diagram of the detailed searching steps for this meta-analysis is described in **Figure 1**. Our search strategy yielded a total of 5,614 studies, of which 1,891 were duplicate entries. We screened 3,723 studies based on the inclusion and exclusion criteria. After a thorough assessment, we excluded 1,855 articles: 895 articles were irrelevant, 581 were meta-analyses and reviews, 174 were conference abstract, and 205 were case reports, short letters, comments, and guidelines. An additional 845 trials were excluded due to a lack of randomized placebo-controlled design and 429 trials were excluded because the subjects were animals. Furthermore, 594 articles were reviewed in more detail, and a full-text screening led to exclusion of 132 duplicated trials. The search strategy yielded 33 randomized placebo-controlled trials that met the inclusion criteria. The Kappa statistic of agreement between the two authors was 87.6%.

### Study Characteristics and Quality Assessment

A total of 37,068 patients received SGLT2i [dapagliflozin (27–35), canagliflozin (36–43), empagliflozin (44–53), sotagliflozin (54, 55) and ertugliflozin (56–58)] and 29,617 patients received placebo. The baseline characteristics of the studies included in this systematic review are shown in **Table 1**. The screening methods of AF/AFL as SAEs are shown in **Table S2** in the **Supplementary Appendix**.

**Table 1:** Baseline information of included studies.

ClinicalTrials.gov number	Published year	Mean age (SD)		Participants	Number of patients			Interventions	Mean follow-up
		Treatment	Control		Treatment	Control	Female (%)		
**Dapagliflozin:**									
NCT03036150 (DAPA-CKD trial) (27)	2020	61.8 (12.1)	61.9 (12.1)	With CKD*	2152	2152	33.1	Dapagliflozin (10 mg) Matching placebo	2.4 years
NCT01730534 (DECLARE-TIMI58 trial) (28)	2019	63.9 (6.8)	64.0 (6.8)	With T2DM who had or were at risk for ASCVD†	8582	8578	37.4	Dapagliflozin (10 mg) Matching placebo	5.2 years
NCT03036124 (DAPA-HF trial) (29)	2019	66.2 (11.0)	66.5 (10.8)	With NYHA class II, III, or IV HF and an EF of 40% or less§	2373	2371	23.4	Dapagliflozin (10 mg) Matching placebo	28.3 months
NCT01646320 (30)	2015	55.2 (8.6)	55.0 (9.6)	With T2DM	160	160	54.4	Dapagliflozin (10 mg) Matching placebo	52 weeks
NCT00528372 (31)	2015	NA‡	NA	With T2DM	483	75	50.5	Dapagliflozin (2.5/5/10 mg) Matching placebo	102 weeks
NCT01042977 (32)	2014	63.9 (7.6)	63.6 (7.0)	With T2DM who had or were at risk for ASCVD	480	482	33.1	Dapagliflozin (10 mg) Matching placebo	24 weeks
NCT01031680 (33)	2013	62.8 (7.0)	63.0 (7.7)	With T2DM, cardiovascular disease and hypertension	455	459	31.7	Dapagliflozin (10 mg) Matching placebo	24 weeks
NCT00528879 (34)	2013	53.7 (NA)	54.0 (NA)	With T2DM	409	137	46.5	Dapagliflozin (2.5/5/10 mg) Matching placebo	102 weeks
NCT00673231 (35)	2012	59.5 (8.1)	58.8 (8.6)	With T2DM	607	193	52.3	Dapagliflozin (2.5/5/10 mg) Matching placebo	24 weeks
**Canagliflozin:**									
NCT02065791(CREDENCE trial) (36)	2019	62.9 (9.2)	63.2 (9.2)	With T2DM and CKD	2202	2199	33.9	Canagliflozin (100 mg) Matching placebo	125 weeks
NCT01032629(CANVAS trial) (37)	2017	62.5 (8.1)	62.3 (7.9)	With T2DM who had or were at risk for ASCVD	2888	1442	33.9	Canagliflozin (100/300 mg) Matching placebo	202 weeks
NCT01989754(CANVAS-R trial) (37)	2017	63.9 (8.4)	64 (8.3)	With T2DM and CKD	2904	2903	37.3	Canagliflozin (100/300 mg) Matching placebo	187 weeks
NCT01064414 (38)	2014	68.7 (8.2)	68.2 (8.4)	With T2DM and CKD	179	90	39.4	Canagliflozin (100/300 mg) Matching placebo	52 weeks
NCT01106651 (39)	2014	63.9 (6.2)	63.2 (6.2)	With T2DM	477	237	44.5	Canagliflozin (100/300 mg) Matching placebo	104 weeks
NCT01022112 (40)	2014	57.3 (10.5)	57.7 (11.0)	With T2DM	308	75	31.9	Canagliflozin (50/100/200/300 mg) Matching placebo	14 weeks
NCT01381900 (41)	2014	56.4 (8.7)	55.8 (9.4)	With T2DM	450	226	46.4	Canagliflozin (100/300 mg) Matching placebo	22 weeks
NCT01106625 (CANTATA-MSU trial) (42)	2013	56.7 (9.7)	56.7 (8.4)	With T2DM	313	156	49	Canagliflozin (100/300 mg) Matching placebo	52 weeks
NCT00642278 (43)	2013	53.1 (8.1)	53.3 (7.8)	With T2DM	321	65	48.7	Canagliflozin (50/100/200/300 mg) Matching placebo	12 weeks
**Empagliflozin:**									
NCT03200860 (EMPA-RESPONSE trial) (44)	2020	79 (73, 83)	73 (61, 83)	With acute decompensated HF	40	39	32.9	Empagliflozin (10 mg) Matching placebo	60 days
NCT03448406 (45)	2020	73.0 (9.0)	73.9 (8.6)	CHF with preserved EF (LVEF > 40%).	157	158	43.2	Empagliflozin (10 mg) Matching placebo	92 days
NCT03448419 (46)	2020	68.7 (9.9)	69.3 (10.6)	CHF with reduced EF (LVEF ≤ 40%)	156	156	25.6	Empagliflozin (10 mg) Matching placebo	91 days
NCT03152552 (47)	2019	68.6 (7.9)	67.8 (10.9)	With T2DM and HF	30	33	38.1	Empagliflozin (25 mg) Matching placebo	36 weeks
NCT01734785 (48)	2016	54.9 (9.7)	55.9 (9.6)	With T2DM	222	110	40.4	Empagliflozin (10/25 mg) Matching placebo	24 weeks
NCT01131676 (EMPA-REG OUTCOME trial) (49)	2016	63.1 (8.6)	63.2 (8.8)	With T2DM	4687	2333	28.5	Empagliflozin (10/25 mg) Matching placebo	5 years
NCT01210001 (50)	2015	54.5 (9.4)	54.6 (10.5)	With T2DM	333	165	51.6	Empagliflozin (10/25 mg) Matching placebo	24 weeks
NCT01011868 (51)	2015	59.2 (10.1)	58.1 (9.4)	With T2DM	324	170	44.1	Empagliflozin (10/25 mg) Matching placebo	82 weeks
NCT01164501 (52)	2014	63.7 (8.9)	64.1 (8.7)	With T2DM and CKD	419	319	41.7	Empagliflozin (10/25 mg) Matching placebo	458 days
NCT00749190 (53)	2013	58.1 (8.6)	59.7 (8.5)	With T2DM	353	71	50.0	Empagliflozin (1/5/10/25/50 mg) Matching placebo	100 days
**Sotagliflozin:**									
NCT02531035 (54)	2020	43.3 (14.1)	42.4 (14.0)	With T1DM	699	703	50.3	Sotagliflozin (400 mg) Matching placebo	28 weeks
NCT02384941 (55)	2019	46.5 (13.3)	45.2 (12.7)	With T1DM	525	268	51.7	Sotagliflozin (200/400 mg) Matching placebo	53 weeks
**Ertugliflozin:**									
NCT01986881 (56)	2020	64.4 (8.1)	64.4 (8.0)	With T2DM who had or were at risk for ASCVD	5499	2747	30.0	Ertugliflozin (5/15 mg) Matching placebo	3.5 years
NCT02033889 (57)	2018	56.7 (8.8)	56.5 (8.7)	With T2DM	412	209	53.6	Ertugliflozin (5/15 mg) Matching placebo	106 weeks
NCT01986855 (58)	2018	67.1 (8.4)	67.5 (8.9)	With T2DM and CKD	313	154	50.5	Ertugliflozin (5/15 mg) Matching placebo	54 weeks

* With an estimated glomerular filtration rate (GFR) of 25 to 75 ml per minute per 1.73 m^2^ of body-surface area and a urinary albumin-to-creatinine ratio (with albumin measured in milligrams and creatinine measured in grams) of 200 to 5000.

† Atherosclerotic cardiovascular disease, defined as clinically evident ischemic heart disease, ischemic cerebrovascular disease, or peripheral artery disease. Participants with multiple risk factors were men 55 years of age or older or women 60 years of age or older who had one or more traditional risk factors, including hypertension, dyslipidemia (defined as a low-density lipoprotein cholesterol level >130 mg per deciliter [3.36 mmol per liter] or the use of lipid-lowering therapies), or use of tobacco.

‡ NA denotes not applicable because the baseline characteristics of the subjects were not available.

§ Eligibility requirements included an age of at least 18 years, an ejection fraction (EF) of 40% or less, and New York Heart Association (NYHA) class II, III, or IV symptoms. Patients were required to have a plasma level of N-terminal pro–B-type natriuretic peptide (NT-proBNP) of at least 600 pg per milliliter (or ≥400 pg per milliliter if they had been hospitalized for HF within the previous 12 months).

**Figure 2:**
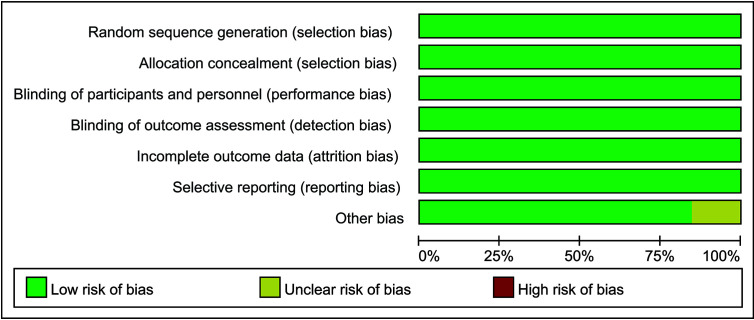
Methodological quality of included studies.

All the included trials had a low risk of bias of random sequence generation (selection bias), allocation concealment, incomplete outcome data, selective reporting bias, except that five trials had unclear other bias. The risk of bias based on the quality of the included trials and the summary of the authors’ judgments of the risk of biases are indicated in **Figure 2**.

### Overall Efficacy Outcome

The SAEs of AF/AFL occurred in 355 of 37,068 patients who were on SGLT2i and 353 of 29,617 patients among those in the placebo group. The SAEs of AF/AFL was significantly lower in SGLT2i group than that of the placebo group (0.96% vs. 1.19%; RR 0.83; 95% CI 0.71–0.96; P = 0.01; I^2^ 25.5%). The SAEs of AF occurred in 291 of 35,464 patients who were on SGLT2i and 298 of 28,229 patients in the placebo group (0.82% vs. 1.06%). The SAEs of AF were significantly lower in the SGLT2i group than that of the placebo group (RR 0.81; 95% CI: 0.69–0.95; P = 0.01; I^2^ 10.2%). Given that the DECLARE-TIMI 58 trial contributed the majority of the patients, sensitivity analysis was performed by excluding this trial. The AF/AFL occurrence were still significantly lower in dapagliflozin group than the placebo group (0.67% vs. 1.13%; RR 0.67; 95% CI 0.46–0.97; P = 0.03; I2 0%), which indicated that the exclusion of DECLARE-TIMI 58 trial did not affect the conclusion at least in the subgroup analysis for dapagliflozin. For all SGLT2i, after we excluded data from the DECLARE-TIMI 58 trial, the risks of AF/AFL incidence between SGLT2i and placebo did not demonstrate statistical significance (0.85% vs. 0.97%; RR 0.87; 95% CI 0.72–1.05; P = 0.16). However, the risks of AF incidence between SGLT2i and placebo was borderline significant (0.73% vs. 0.90%; RR 0.83; 95% CI 0.67–1.01; P = 0.07).

### Subgroup Outcome

**Figure 3:**
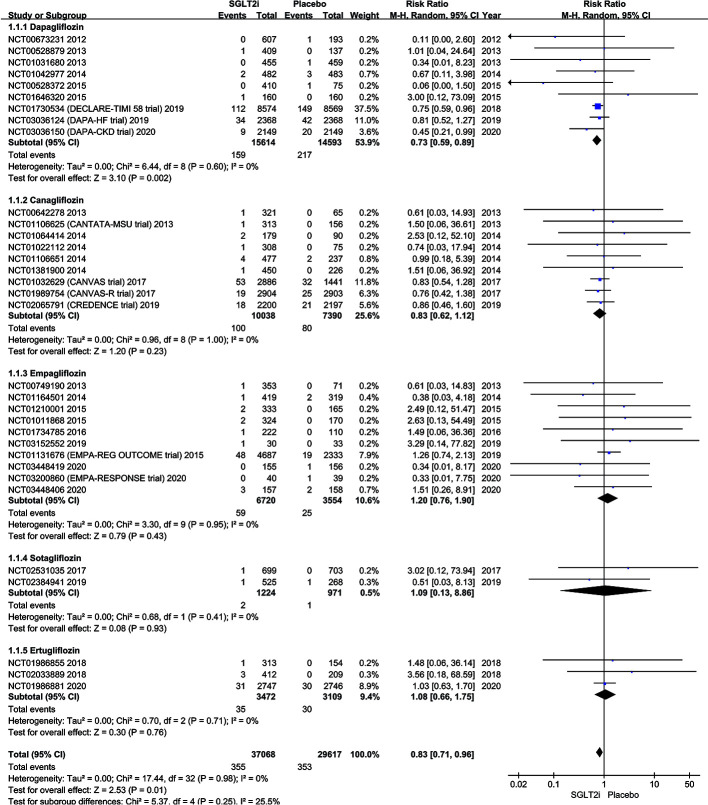
Forest plot comparing AF/AFL occurrence between SGLT2 inhibitors group and placebo group.

A subgroup analysis of studies comparing SGLT2i (dapagliflozin, canagliflozin, empagliflozin, sotagliflozin, and ertugliflozin) with the placebo group is shown in **Figure 3**. Altogether, 159 of 15,614 patients in the dapagliflozin group had SAEs of AF/AFL in comparison to a higher incidence of 217 out of 14,593 for the placebo group (1.02% vs. 1.49%; RR 0.73; 95% CI 0.59–0.89; P = 0.002; I^2^ 0%). Though the SAEs incidence of AF/AFL was lower in the canagliflozin group compared with those on placebo, the canagliflozin and placebo groups did not differ in the incidence of AF/AFL (1.00% vs. 1.08%; RR 0.83; 95% CI 0.62–1.12; P = 0.23; I^2^ 0%). Also, there was no difference in the SAEs of AF/AFL in the empagliflozin group (0.88% vs. 0.70%; RR 1.20; 95% CI 0.76–1.90; P = 0.43; I^2^ 0%), sotagliflozin group (0.16% vs. 0.10%; RR 1.09; 95% CI 0.13–8.86; P = 0.93; I^2^ 0%) and ertugliflozin (1.01% vs. 0.96%; RR 1.08; 95% CI 0.66–1.75; P = 0.76; I^2^ 0%) versus matching placebo. **Figure 3** illustrates the comparison of AF/AFL events. The symmetrical funnel plot suggests no significant publication bias (**Figure S1** in the **Supplementary Appendix**).

**Figure 4:**
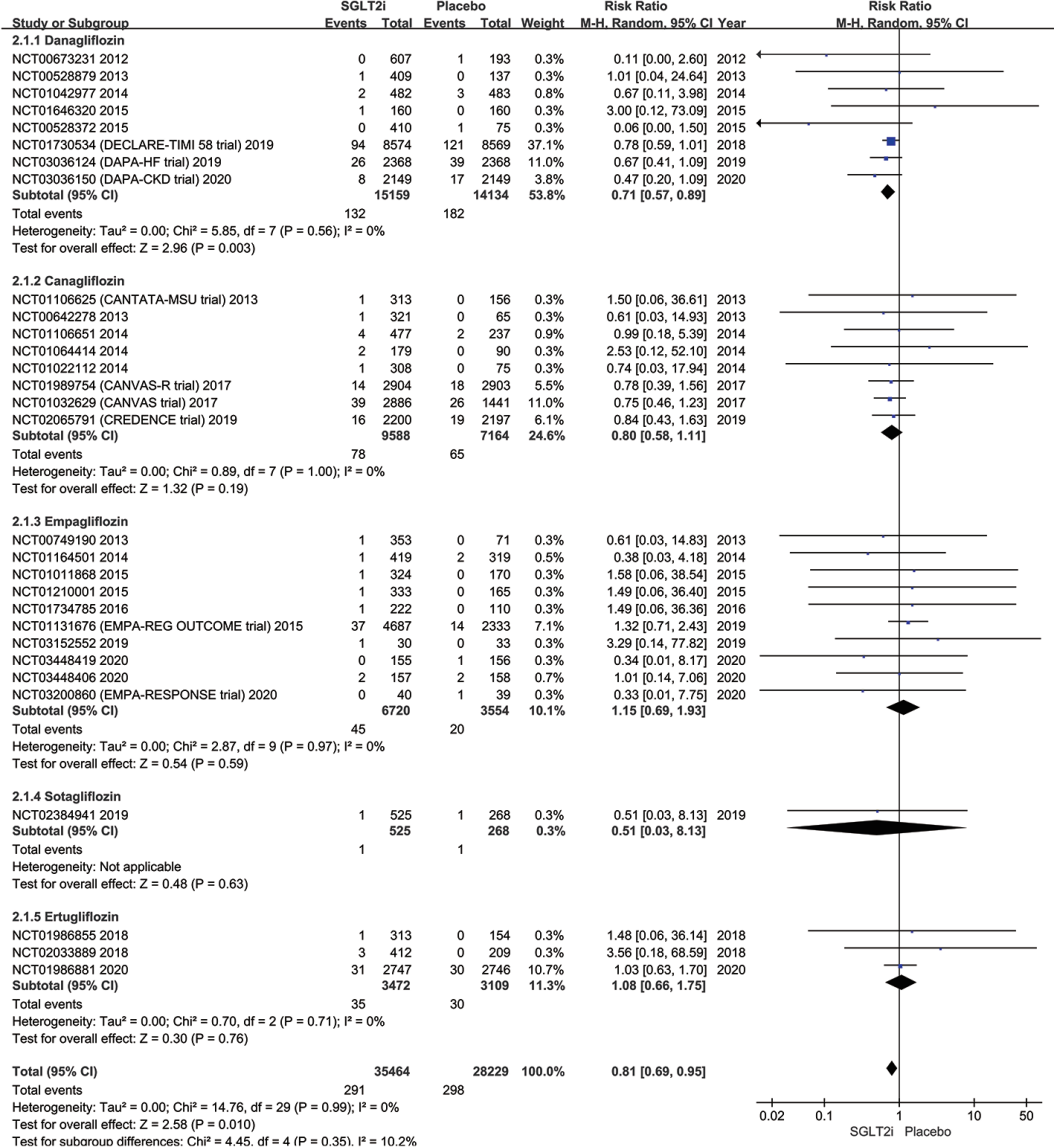
Forest plot comparing AF occurrence between SGLT2 inhibitors group and placebo group.

The subgroup analysis of all 33 trials reported the outcomes of 291 SAEs of AF among all SGLT2i classes are given in **Figure 4**. The SAEs of AF occurred in 132 of 15,159 patients in dapagliflozin group and 182 of 14,134 placebo (0.87% vs. 1.29%; RR 0.71; 95% CI 0.57–0.89; P = 0.003; I^2^ 0%). The pooled SAEs incidence of AF was also lower in the canagliflozin group compared with placebo with no significant difference in the SAEs incidence of AF in the two groups (0.81% vs. 0.91%; RR 0.80; 95% CI 0.58–1.11; P = 0.19; I^2^ 0%). Similarly, there was no significant difference between empagliflozin group (0.67% vs. 0.56%; RR 1.15; 95% CI 0.69–1.93; P = 0.59; I^2^ 0%), sotagliflozin (0.19% vs. 0.37%; RR 0.51; 95% CI 0.03–8.13; P = 0.63; I^2^ not applicable) or ertugliflozin (1.01% vs. 0.96%; RR 1.08; 95% CI 0.66–1.75; P = 0.76; I^2^ 0%) compared to placebo. A symmetrical funnel plot suggests that there was no significant publication bias (**Figure S2** in the **Supplementary Appendix**).

## Discussion

This systematic review and meta-analysis analyzed AF/AFL of approximately 66,685 patients who were on either SGLT2i or placebo. Our results show that SGLT2i was associated with a lower incidence of AF/AFL. The pooled incidence of AF/AFL was 19.33% lower in SGLT2i compared with those on placebo (0.96% vs. 1.19%), with a significant difference in the risk of AF/AFL between the two groups. In the subgroup analysis of the individual SGLT2i drugs, dapagliflozin was associated with an approximate 31.54% lower SAEs of AF/AFL incidence favoring the SGLT2i group over the placebo group. However, there were no significant differences in the incidence of AF/AFL in the other members of the SGLT2i class, namely canagliflozin, empagliflozin, sotagliflozin, and ertugliflozin when compared to placebo.

The results of this meta-analysis are relevant for the growing body of patients who are on SGLT2i especially for T2DM with diabetes-related comorbidities or cardiovascular death risk factors. This systematic review and meta-analysis shows that SGLT2i may reduce AF/AFL occurrence. More specifically, dapagliflozin decreased the incidence of reported SAEs of AF/AFL. According to the CVD-REAL 2 Study (59) and a consensus report by the American Diabetes Association (ADA) and the European Association for the Study of Diabetes (EASD) (60), SGLT2i contributes a beneficial effect in reducing cardiovascular comorbidities. Recently, a post-hoc analysis based on the DECLARE-TIMI58 trial found that dapagliflozin lowered the incidence of reported episodes of AF/AFL in high-risk T2DM patients (20). Also, the findings of our meta-analysis buttress the findings of the DAPA-HF and DAPA-CKD trials (22). The ongoing DELIVER trial, a study designed to detect the therapeutic effects of dapagliflozin in HF with preserved ejection fraction (61), is expected to further explain the relationship between AF/AFL and HF in patients without diabetes mellitus.

In this meta-analysis, canagliflozin, empagliflozin, sotagliflozin, and ertugliflozin do not appear to reduce the incidence of reported SAEs of AF/AFL. Canagliflozin use was initially found to reduce AF/AFL incidence in individual studies but was no longer statistically significant in the meta-analysis. This is likely due to the relatively small sample size and low AF/AFL incidence rate. The influence of canagliflozin on AF/AFL occurrence warrants further investigation. Interestingly, in the EMPA-REG OUTCOME trial (49), despite seeing an improvement in HF, patients in the empagliflozin arm were found to have an increased SAEs of AF/AFL incidence.

The post-hoc analysis of the DECLARE-TIMI 58 trial found dapagliflozin reduced the effect of AF/AFL incidence was irrespective of baseline AF/AFL. Moreover, the presence of atherosclerotic cardiovascular disease versus multiple risk factors or a history of HF did not alter the reduction in AF/AFL events. Further, there were no interaction effects with respect to gender, prior ischemic stroke, HbA1c levels, body mass index, blood pressure or estimated glomerular filtration rate. In a recent meta-analysis, Li et al. indicated that the AF/AFL reduction benefit of SGLT2i have no relevance with age, body weight, and systolic blood pressure at baseline (26). In another meta-analysis, Okunrintemi et al. considered the AF/AFL reduction may be associated with decreased uric acid and increased magnesium induced by SGLT2i (25). Moreover, the protective effects of SGLT2i against AF/AFL may be direct actions on cardiac remodeling by reducing oxidative stress (62), which can prevent mitochondrial dysfunction and improve mitochondrial energetics (63). Thus, the current evidence points toward both a systematic and cardio-specific mechanism in preventing arrhythmias (64), while the relative contributions from either pathway remain unclear. The clinical significance our finding is that SGLT2i may reduce mortality, incident HF and HF-related hospitalizations at least partly by reducing AF/AFL occurrences. However, mediation analysis is needed to confirm this. Whether SGLT2i use is associated with a reduced incidence of ischemic stroke remains to be elucidated in future studies.

Previous evidence has confirmed a lower AF incidence with ACEIs and ARBs (65–67). Within the context of T2DM, hypoglycemic medications were rarely reported to reduce the incidence of AF. Published placebo-controlled clinical trials are known to be less susceptible to selection and recall bias compared with observational studies. As such, the latter studies were excluded from the meta-analysis. Recently, two meta-analyses on the relationship between SGLT2i use and AF outcomes have been performed (25, 26). Our current meta-analysis extends these two studies by including the largest number of trials (n = 33) involving 66,685 patients.

### Limitations

In this meta-analysis, several limitations can be stated. Firstly, the AF/AFL incidence in the included studies was relatively low, and the AF/AFL incidence was calculated by reported SAEs among trials. This may have underestimated the reported pooled incidence rate of AF/AFL. Secondly, it should be acknowledged that there were differences in baseline characteristics of the patients, such as follow-up duration, sample size, age, and gender. Only the DECLARE-TIMI 58 trial was the most inclusive cardiovascular outcomes trial, with a broad representation of patients encountered in routine clinical practice than those of CANVAS and EMPA-REG OUTCOME (68). Thirdly, our meta-analysis demonstrated intermediate heterogeneity among SGLT2i, although subgroup analysis demonstrated no significant heterogeneity. Fourthly, the dose of some SGLT2i (canagliflozin and empagliflozin) was not consistent between the trials, thus the potential dose-reaction effect may influence the AF/AFL incidence. Finally, few randomized placebo-controlled trials have reported the findings for individual SGLT2i drugs apart from dapagliflozin, thus the potential benefits of these drugs on AF/AFL remain to be elucidated.

## Conclusions

The use of SGLT2i is associated with a 19.33% lower risk of AF/AFL compared with the placebo. Dapagliflozin users had the lowest risk of AF/AFL episodes.

## Data Availability Statement

The original contributions presented in the study are included in the article/**Supplementary Material**. Further inquiries can be directed to the corresponding author.

## Author Contributions

This study was conceived and designed by XY. YL and YWa were responsible for data collection and data analysis. DL wrote the main manuscript text. TH and XY supervised data collection and data analysis. All authors contributed to the article and approved the submitted version.

## Funding

Supported by Chang Jiang Scholars Program (T2017124) from Ministry of Education, the People’s Republic of China, the Program of Liaoning Distinguished Professor for YX, and National Natural Science Foundation of China (81970286) for YX.

## Conflict of Interest

The authors declare that the research was conducted in the absence of any commercial or financial relationships that could be construed as a potential conflict of interest.

## Abbreviations

ACEIs, angiotensin-converting enzyme inhibitors; ADA, American Diabetes Association; AF, atrial fibrillation; AFL, atrial flutter; ARBs, angiotensin receptor blockers; CANVAS, Canagliflozin Cardiovascular Assessment Study; CVD-REAL, comparative effectiveness of cardiovascular outcomes in new users of SGLT-2 inhibitors; DAPA-HF, dapagliflozin in patients with heart failure and reduced ejection fraction; DAPA-CKD, dapagliflozin in patients with chronic kidney disease; DECLARE-TIMI 58, dapagliflozin effect on cardiovascular events–thrombolysis in myocardial infarction 58; DELIVER, dapagliflozin evaluation to improve the LIVEs of patients with preserved ejection fraction heart failure; EASD, European Association for the Study of Diabetes; EMPA-REG OUTCOME, empagliflozin, cardiovascular outcomes, and mortality in type 2 diabetes; HF, heart failure; RR, risk ratio; SAEs, serious adverse events; SGLT2i, sodium-glucose transporter 2 inhibitor; T2DM, type 2 diabetes mellitus.
