# Supplementary material for: Protective Effects of Sodium-Glucose Transporter 2 Inhibitors on Atrial Fibrillation and Atrial Flutter: A Systematic Review and Meta- Analysis of Randomized Placebo-Controlled Trials
Source: Front Endocrinol (Lausanne). 2021 Mar 19;12:619586. doi: 10.3389/fendo.2021.619586 (PMC8018283; doi:10.3389/fendo.2021.619586)
Supplement: Supplementary file 1 [file DataSheet_1.docx]

Supplementary Appendix

**Figure S1 | Funnel plot of standard errors against logarithms of risk ratios for studies comparing AF/AFL occurrence between SGLT2 inhibitors group and placebo group.**


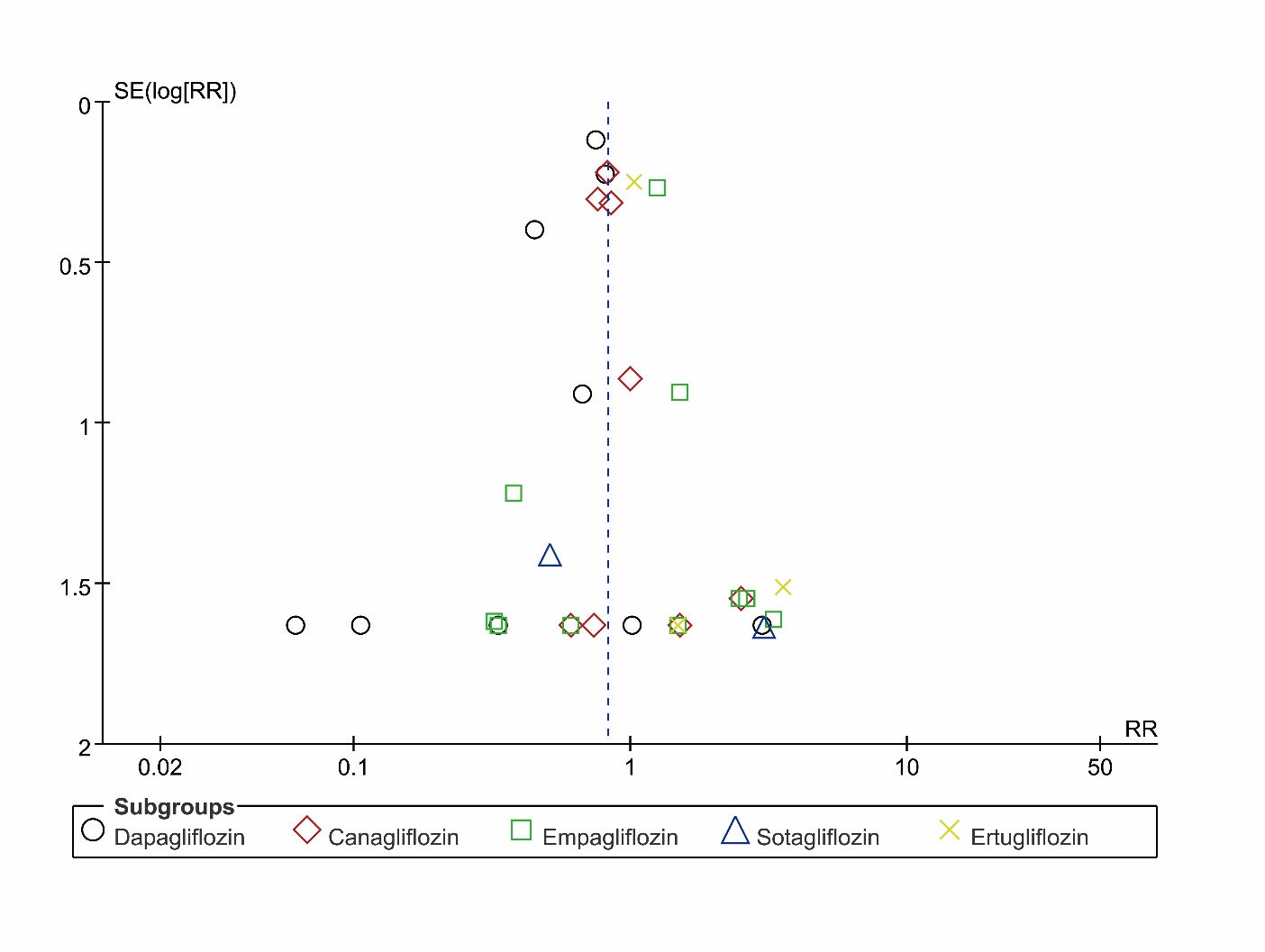


**Figure S2 | Funnel plot of standard errors against logarithms of risk ratios for studies comparing AF occurrence between SGLT2 inhibitors group and placebo group.**

**
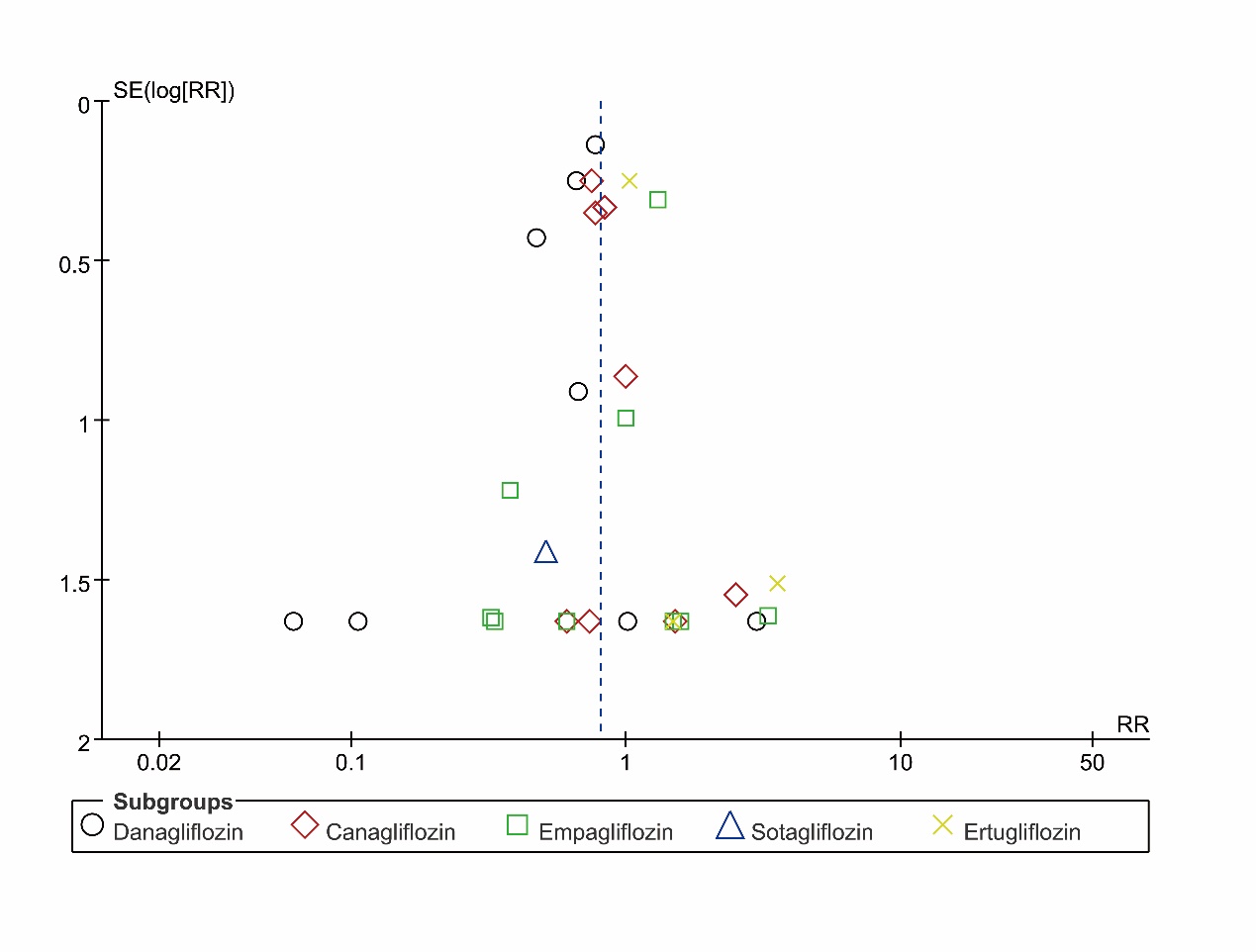
**

Table S1 | The search algorithm of systematic review and meta-analysis (till December 3, 2020).

| PubMed | (((((((("Sodium-Glucose Transporter 2 Inhibitors"[Mesh]) OR "2-(3-(4-ethoxybenzyl)-4-chlorophenyl)-6-hydroxymethyltetrahydro-2H-pyran-3,4,5-triol" [Supplementary Concept]) OR "Canagliflozin"[Mesh]) OR "empagliflozin" [Supplementary Concept]) OR "(2S,3R,4R,5S,6R)-2-(4-chloro-3-(4-ethoxybenzyl)phenyl)-6-(methylthio)tetrahydro-2H-pyran-3,4,5-triol" [Supplementary Concept]) OR "6-((4-ethylphenyl)methyl)-3',4',5',6'-tetrahydro-6'-(hydroxymethyl)spiro(isobenzofuran-1(3H),2'-(2H)pyran)-3',4',5'-triol" [Supplementary Concept]) OR "1,5-anhydro-1-(5-(4-ethoxybenzyl)-2-methoxy-4-methylphenyl)-1-thioglucitol" [Supplementary Concept]) OR "ipragliflozin" [Supplementary Concept]) OR "ertugliflozin" [Supplementary Concept])  Filters applied: Clinical Trial |
| --- | --- |
| Cochrane | (((((((((((((((("Sodium-Glucose Transporter 2 Inhibitors"[Mesh]) OR "kw: dapagliflozin") OR “BMS 512148”) OR "Canagliflozin"[Mesh]) OR "kw: empagliflozin") OR "BI 10773”) OR "kw: luseogliflozin") OR "TS071") OR "kw: tofogliflozin") OR "CSG452") OR "kw: ipragliflozin") OR "ASP1941") OR "kw: sotagliflozin") OR "LX4211" OR "kw: ertugliflozin") OR "PF 04971729")  Filters applied: Trials |
| Web of Science | TS: ("sodium-glucose transporter 2 inhibitors") OR TS: (“sodium-glucose cotransporter 2 inhibitors”) OR TS: ("SGLT2 inhibitors") OR TS: (SGLT2i) OR TS: (dapagliflozin) OR TS: (BMS 512148) OR TS: (empagliflozin) OR TS: (BI 10773) OR TS: (canagliflozin) OR TS: (JNJ 28431754) OR TS: (lpragliflozin) OR TS: (ASP1941) OR TS: (luseoglifozin) OR TS: (TS071) OR TS: (tofogliflozin) OR TS: (CSG452) OR TS: (sotaglifozin) OR TS: (LX4211) OR TS: (ertugliflozin) OR TS: (PF 04971729) AND ALL (“clinical trial”) |
| Scopus | (KEY ("sodium-glucose transporter 2 inhibitors") OR KEY (“sodium-glucose cotransporter 2 inhibitors”) OR KEY ("SGLT2 inhibitors") OR KEY (SGLT2i) OR KEY (dapagliflozin) OR TITLE-ABS-KEY (BMS 512148) OR KEY (empagliflozin) OR TITLE-ABS-KEY (BI 10773) OR KEY (canagliflozin) OR TITLE-ABS-KEY (JNJ 28431754) OR KEY (lpragliflozin) OR TITLE-ABS-KEY (ASP1941) OR KEY (luseoglifozin) OR TITLE-ABS-KEY (TS071) OR KEY (tofogliflozin) OR TITLE-ABS-KEY (CSG452) OR KEY (sotaglifozin) OR TITLE-ABS-KEY (LX4211) OR KEY (ertugliflozin) OR TITLE-ABS-KEY (PF 04971729) AND ALL (“clinical trial”)) AND (LIMIT-TO (SUBJAREA, “MEDI”)) |

**Table S2 | The screening methods of AF/AFL.**

| **ClinicalTrials.gov number** | **The diagnosis of AF/AFL** |
| --- | --- |
|  |  |
| **Dapagliflozin:** |  |
| NCT03036150 (DAPA-CKD trial) | Not given. |
| NCT01730534 (DECLARE-TIMI58 trial) | Not given. |
| NCT03036124 (DAPA-HF trial) | A 12-lead electrocardiogram (standard ECG with a paper speed of 25-50 mm/second covering at least 6 sequential beats) will be recorded after the patient has been lying down to rest for at least 5 minutes. ECG date, heart rate and heart rhythm will be recorded. Pulse will be measured three times at all visits. |
| NCT01646320 | Not given. |
| NCT00528372 | Not given. |
| NCT01042977 | Not given. |
| NCT01031680 | Not given. |
| NCT00528879 | Not given. |
| NCT00673231 | Not given. |
| **Canagliflozin:** |  |
| NCT02065791(CREDENCE trial) | Not given. |
| NCT01032629(CANVAS trial) | The ECG variables that will be analyzed are heart rate, PR interval, QRS interval, QT interval, and QTc using the Fridericia (QTcF) correction methods. |
| NCT01989754(CANVAS-R trial) | Not given. |
| NCT01064414 | Not given. |
| NCT01106651 | 12-lead ECG |
| NCT01022112 | 12-lead ECG |
| NCT01381900 | Not given. |
| NCT01106625 (CANTATA-MSU trial) | Not given. |
| NCT00642278 | Not given. |
| **Empagliflozin:** |  |
| NCT03200860 (EMPA-RESPONSE trial) | ECG and Holter ECG monitoring |
| NCT03448406 | Outcomes of ECGs will be part of the reporting of medical history or AE reporting. |
| NCT03448419 | Outcomes of ECGs will be part of the reporting of medical history or AE reporting. |
| NCT03152552 | The following variables will be descriptively summarized by visit and treatment: QTcF duration (msec), heart rate (beats/min), PR duration (msec), RR duration (msec), and QRS duration (msec). |
| NCT01734785 | Not given. |
| NCT01131676 (EMPA-REG OUTCOME trial) | All ECGs will be collected on standardised devices and stored at a central digital database provided by a central ECG service. At each visit as indicated in the flowchart and in addition in case of cardiac symptoms (indicating rhythm disorders or cardiac ischaemia) or upon suspicion of cardiovascular events, 12-lead ECGs (I, II, III, aVR, aVL, aVF, V1 - V6) will be recorded according to flowchart. The ECGs will be evaluated by the study investigator or a designate for immediate treatment. In addition, the central ECG vendor will provide manual reading results which will be communicated to the investigator for his further judgment and will be stored in the central database of the vendor. Additional ECGs recorded outside the investigator's facilities may be collected by the investigator and transferred to the central database for safety reasons. Upon receipt of an alert from the ECG vendor, the investigator or designate is expected to review, sign and date the findings, record their clinical judgement in the source documents. Agreement with the findings should result in a reported (S)AE if judged to be clinically relevant. If the investigator does not agree with the ECG reviewer’s findings, a note should be made in the source regarding the review of the alert. Clinically relevant changes in the ECG will be reported as AEs. |
| NCT01210001 | Not given. |
| NCT01011868 | Not given. |
| NCT01164501 | Not given. |
| NCT00749190 | Not given. |
| **Sotagliflozin:** |  |
| NCT02531035 | The ECG Core Laboratory will review protocol-scheduled ECGs to assess the presence of Q-waves (ie, potential “silent” MI) and/or left bundle branch block (LBBB). The assessments recorded on the final “outcome” ECG Core Laboratory Assessment Forms will be used for the analysis. |
| NCT02384941 | Not given. |
| **Ertugliflozin:** |  |
| NCT01986881 | A centrally read 12-lead ECG was collected at baseline, week 18, week 52, and annually thereafter. |
| NCT02033889 | Not given. |
| NCT01986855 | Not given. |
